# Efficient protocol of *de novo* shoot organogenesis from somatic embryos for grapevine genetic transformation

**DOI:** 10.3389/fpls.2023.1172758

**Published:** 2023-05-31

**Authors:** Luca Capriotti, Angela Ricci, Barbara Molesini, Bruno Mezzetti, Tiziana Pandolfini, Irene Piunti, Silvia Sabbadini

**Affiliations:** ^1^ Department of Agricultural, Food and Environmental Sciences, Marche Polytechnic University, Ancona, Italy; ^2^ Department of Biotechnology, University of Verona, Verona, Italy

**Keywords:** *Vitis vinifera*, somatic embryogenesis, *Agrobacterium tumefaciens*, *in vitro* organogenesis, transformation efficiency

## Abstract

Plant genetic transformation is a powerful tool that can facilitate breeding programs for disease tolerance, abiotic stress, fruit production, and quality by preserving the characteristics of fruit tree elite genotypes. However, most grapevine cultivars worldwide are considered recalcitrant, and most available genetic transformation protocols involve regeneration by somatic embryogenesis, which often requires the continuous production of new embryogenic calli. Cotyledons and hypocotyls derived from flower-induced somatic embryos of the Vitis vinifera cultivars Ancellotta and Lambrusco Salamino, in comparison with the model cultivar Thompson Seedless, are here validated for the first time as starting explants for *in vitro* regeneration and transformation trials. Explants were cultured on two different MS-based culture media, one having a combination of 4.4 µM BAP and 0.49 µM IBA (M1), and the other only supplemented with 13.2 µM BAP (M2). The competence to regenerate adventitious shoots was higher in cotyledons than in hypocotyls on both M1 and M2. M2 medium increased significantly the average number of shoots only in Thompson Seedless somatic embryo-derived explants. This efficient regeneration strategy, that proposes a combination of somatic embryogenesis and organogenesis, has been successfully exploited in genetic engineering experiments. Ancellotta and Lambrusco Salamino cotyledons and hypocotyls produced the highest number of calli expressing eGFP when cultured on M2 medium, while for Thompson Seedless both media tested were highly efficient. The regeneration of independent transgenic lines of Thompson Seedless was observed from cotyledons cultured on both M1 and M2 with a transformation efficiency of 12 and 14%, respectively, and from hypocotyls on M1 and M2 with a transformation efficiency of 6 and 12%, respectively. A single eGFP fluorescent adventitious shoot derived from cotyledons cultured on M2 was obtained for Ancellotta, while Lambrusco Salamino showed no regeneration of transformed shoots. In a second set of experiments, using Thompson Seedless as the model cultivar, we observed that the highest number of transformed shoots was obtained from cotyledons explants, followed by hypocotyls and meristematic bulk slices, confirming the high regeneration/transformation competences of somatic embryo-derived cotyledons. The independent transformed shoots obtained from the cultivars Thompson Seedless and Ancellotta were successfully acclimatized in the greenhouse and showed a true-to-type phenotype. The novel *in vitro* regeneration and genetic transformation protocols optimized in this study will be useful for the application of new and emerging modern biotechnologies also to other recalcitrant grapevine genotypes.

## 1 Introduction

Not all plant cells have the same regeneration competence, and the genetic factors, as well as the environmental factors, appear to be preponderant to give rise to *de novo* adventitious growth (Fehér, 2015). Higher plants, possess meristematic areas in the root and shoot apices that retain embryonic activity through unlimited cell division, and then differentiate into cells responsible for the growth of the plant primary and secondary structures (Murray et al., 2012; Kane, 2018). This innate plasticity competence is the basis of *in vitro* culture, which has been widely employed in plant mass propagation and biotechnology, as well as to perform functional genetic studies. Regeneration of plants from somatic tissues, after the first stage of pluripotency acquisition, is generally feasible through *de novo* shoot organogenesis or somatic embryogenesis, both highly dependent on tissue type and external auxin and cytokinin signals (Ikeuchi et al., 2016). During the process of organogenesis, cells acquire the competence to give rise to a specific meristem (i.e., the shoot apical meristem), and then later regenerate the missing portion (i.e., the root system); while somatic embryogenesis allows the simultaneous development of both the caulinar and radical poles in the new regenerated individual. In *Vitis* spp., although *de novo* shoot organogenesis has been successfully reported using different organs as starting explants (leaf segments, *in vitro* leaves, internodes, petioles, apical and lateral meristems), the number of reports describing the use of this regeneration process to obtain genetically transformed lines are limited (Xie et al., 2016; Zhang et al., 2021). The highest transformation efficiencies (from 10% up to almost 50%) have been reported in more than one *Vitis* genotype, including the table grape cultivar Thompson Seedless, by using slices of a vegetative structure called meristematic bulk (MB), resulting from the mechanical treatment and repeated subcultures of meristematic shoot apices in media with increasing cytokinin concentrations (Mezzetti et al., 2002; Xie et al., 2016; Sabbadini et al., 2019a; Sabbadini et al., 2019b). What these studies has in common is the strong dependence of the success of genetic transformation on the genotype used. Some cultivars such as Thompson Seedless and Chardonnay were efficient in regenerating new independent transformed lines, whereas other genotypes were unable to regenerate shoots from transformation events (Xie et al., 2016; Sabbadini et al., 2019b). Typically, transformation events, especially during the first phases after *Agrobacterium tumefaciens* infection, involve few plant cells, depending on the genotype recalcitrance. Therefore, an effective selection and regeneration strategy is essential to identify the few transgenic cells among those that have not introgressed the gene of interest, and to obtain transgenic plants in the later steps (Delporte et al., 2014). Hence, most genetic transformations in grapevine have been performed on embryogenic calli or somatic embryos as starting explants induced from the most disparate somatic tissues (Dhekney et al., 2019; Zhang et al., 2021; Nuzzo et al., 2022). There is no unique developmental pathway to obtain somatic embryos, indeed, it has been demonstrated that somatic embryo formation can occur directly or indirectly from a starting explant, and the entire process can be initiated from differentiated somatic cells or pericycle-like stem cells (Fehér, 2019; Capriotti et al., 2022). Recent publications highlighted the importance to have well-established somatic embryogenesis regeneration protocols for the application of “new genomic techniques” (NGTs) on additional cultivars and rootstocks of Italian and international interest (Forleo et al., 2021; Capriotti et al., 2022; Nerva et al., 2023).

However, one of the major limitations of this regeneration technique regards embryo endodormancy, resulting in low germination rate and plant development (Gray and Purohit, 1991; Larrouy et al., 2017). Variable germination rates, often lower than 50%, were reported in somatic embryogenesis studies (Martinelli et al., 2001b; Carimi et al., 2005). Jayasankar and co-authors demonstrated that somatic embryos obtained on solid culture exhibited physiological dormancy and a different morphology than those obtained in liquid medium, enhancing the germination rate by more than 40% (Jayasankar et al., 2003). Vilaplana and Mullins (1989) described an efficient protocol for regenerating adventitious shoots in different cultivars of grapevine, *via* organogenesis, starting from cotyledons and hypocotyls obtained from flower-derived somatic embryos. The results achieved showed the overcoming of the embryo-plantlet conversion stage, leading to an higher regeneration capacity of these explants compared to intact somatic embryos (Vilaplana and Mullins, 1989). Adventitious shoot regeneration, *via* organogenesis, starting from grapevine somatic embryos-derived cotyledons was also successfully obtained by Martinelli et al. (2001b).

The development of efficient *in vitro* regeneration protocols is the base for the application of genetic transformation techniques in plants. Since these protocols are genotype-dependent, it is important to identify the most efficient regeneration and transformation protocols for grapevine cultivars of local interest, such as Lambrusco Salamino and Ancellotta. In previous experimental trials carried out on these Italian grapevine cultivars by our research group, the direct transformation of somatic embryos or somatic embryogenic calli, by following the protocol described by Dhekney et al. (2016), was unsuccessful (unpublished results).

In this study, we optimized a regeneration protocol, based on the method described by Vilaplana and Mullins (1989), that led to efficient adventitious shoot formation, *via* organogenesis, from cotyledons and hypocotyls derived from somatic embryos. These protocols have also been exploited to carry out genetic transformation trials. The regeneration and transformation potential of these Italian *Vitis vinifera* cultivars, Ancellotta and Lambrusco Salamino, has been compared with that of Thompson Seedless, which is the main reference genotype for grapevine, having shown high transformation efficiencies by applying different protocols (Li et al., 2006; Xie et al., 2016; Sabbadini et al., 2019b). The use of cotyledons and hypocotyls, in comparison with the MB regeneration system, allowed to increase the number of transformed lines just nine weeks after *Agrobacterium* infection in Thompson Seedless, and to obtain the first transformed plant for Ancellotta, for which genetic transformation had not been demonstrated until now.

## 2 Materials and methods

### 2.1 Plant material and culture conditions

**Figure 1:**
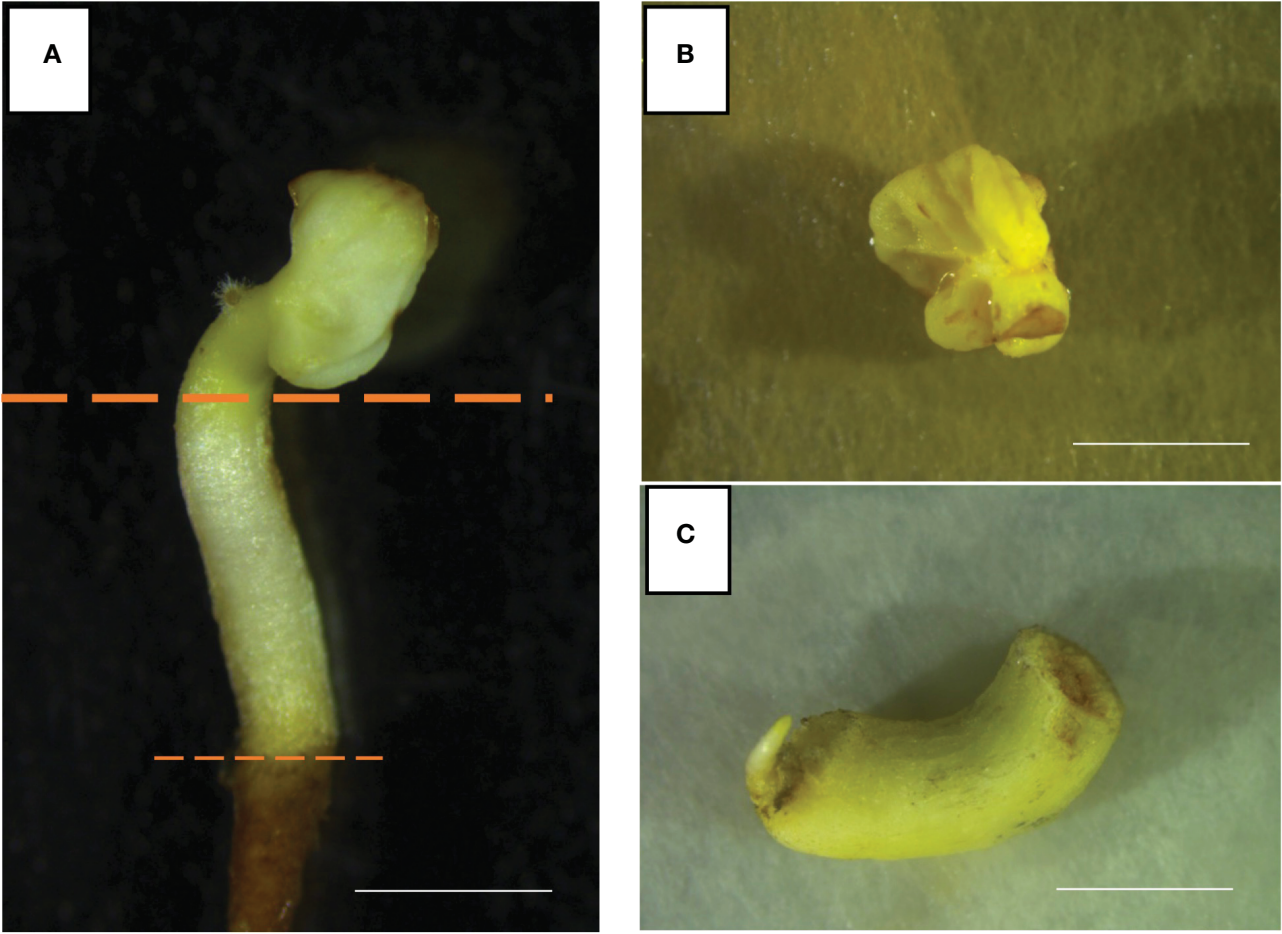
Well-developed somatic embryos of Ancellotta cultivar **(A)** were dissected separating cotyledons **(B)**, and hypocotyls **(C)** (*bars* = 5 mm).

Well-developed somatic embryos of the *Vitis vinifera* cultivars Ancellotta, Lambrusco Salamino, and Thompson Seedless were used as source of starting explants (**Figure 1A**). These explants derive from the development of embryogenic calli induced from whole flowers, stamens, and pistils of the respective *Vitis vinifera* cultivars, following the protocols optimized by Capriotti et al., 2022. In sterile conditions, mature somatic embryos (SE) at the cotyledonary stage were sliced, separating hypocotyls from cotyledons, and discarding the primary radicle (**Figure 1A**). The two almost-united cotyledons (**Figure 1B**) and the hypocotyls (**Figure 1C**) were placed on 90 mm-Petri dishes, filled with two different novel shoot regeneration media, which consisted of Murashige and Skoog (MS) (Murashige and Skoog, 1962) basal salts and vitamins supplemented with 15 g L^−1^ sucrose, 7 g L^−1^ plant agar (Duchefa Biochemie, Haarlem, The Netherlands). This basal medium was supplemented with two specific combinations of plant growth regulators (PGRs) to induce shoot regeneration from cotyledons and hypocotyls. Medium 1 (M1) was supplemented with 4.4 μM 6 Benzyl-aminopurine (BAP) and 0.49 μM 3-Indole-butyric acid (IBA) (Vilaplana and Mullins, 1989; Martinelli et al., 2001b), and medium 2 (M2) with 13.2 μM BAP, the latter selected based on the previous experience described using the MB regeneration system (Sabbadini et al., 2019b). The pH was adjusted to 5.7 using 1N KOH solution, and the medium was autoclaved at 121°C for 20 minutes.

A total of 50 cotyledons and 50 hypocotyls from each of the *Vitis vinifera* cultivars Ancellotta, Lambrusco Salamino, and Thompson Seedless, divided into five replicates (ten explants for each Petri plate), for each culture medium, were positioned on 90 mm-Petri dishes, containing around 25 mL of culture medium, sealed with parafilm, and placed in the growth chamber at 24 ± 1°C under a photoperiod of 16-h light (70 µmol m**^-^** ^2^ s**^-^** ^1^), provided by white fluorescent tubes. The explants were transferred on fresh media every 3 weeks for a total of three subcultures. The regeneration experiment was replicated three times. Regeneration responses of cotyledons and hypocotyls on each medium and for each genotype were monitored, recording at the ninth week the percentage of regenerating explants [(number of explants regenerating shoots/total explants treated) × 100] and the average number of shoots calculated exclusively on non-necrotic explants. All the data were recorded after 9 weeks of culture under the same culture conditions.

### 2.2 *Agrobacterium-mediated* transformation and cotyledons and hypocotyls transformation conditions

*Agrobacterium tumefaciens* strain EHA105 carrying the binary vector pK7WG2 was used in this study. The T-DNA of the binary vector contains the selection marker *nptII* gene, which confers resistance to kanamycin and the reporter *green fluorescent protein* (*eGFP*) gene (Sabbadini et al., 2019b). SEs at the cotyledonary stage selected from three-to four-week-old SE cultures on X6 medium were sliced, separating cotyledons and hypocotyls, and placed on fresh X6 medium. A single colony of the engineered *A. tumefaciens* was inoculated into 10 mL of liquid Luria Bertani medium (LB) containing 60.76 µM rifampicin, and 225.67 µM spectinomycin, and incubated overnight at 28°C with constant agitation (175 rpm), reaching an O.D. at 600 nm between 0.5-1. Bacterial cultures were centrifuged at 4200 x g for 8 minutes at room temperature, and the bacterial pellet was resuspended in 20 mL of liquid X2 medium (Dhekney et al., 2016), together with 100 µM acetosyringone, and mixed on a rotary shaker at 180 rpm at 24°C for 3 hours. A total of 50 cotyledons and 50 hypocotyls from each of the *Vitis vinifera* cultivar Ancellotta, Lambrusco Salamino, and Thompson Seedless, for each culture medium under study, were immersed in the resuspended *Agrobacterium* inoculum keeping them separately in falcon tubes for 15 minutes at 24°C, covering the falcons with aluminum foil. Explants were blotted dry and placed in 90 mm-Petri dishes containing antibiotic-free X2 solid medium having at the top two filter papers previously soaked with a solution of X2 and 100 µM acetosyringone, and co-cultured for 48 hours at 24°C in dark condition. At the end of the co-culture, the SEs were washed for 1 hour in a sterile distilled water solution supplemented with 420 µM cefotaxime and 475 µM carbenicillin. Explants were blotted dry and cotyledons were placed with the abaxial face in contact with the culture media, while hypocotyls were cultured horizontally in microboxes (Micropoli, IT) filled with two different regeneration and selective media containing MS medium (Murashige and Skoog, 1962), and including 30 g L^−1^ sucrose, 7 g L^−1^ plant agar, 146 µM kanamycin, 420 µM cefotaxime, and 475 µM carbenicillin. Two phytohormones combination were used to induce shoot regeneration: one with a combination of 4.4 µM BAP and 0.49 µM IBA (selective M1), and the other that includes 13.2 µM BAP (selective M2). *De novo* shoot organogenesis occurred under light provided by white fluorescent tubes (16 hours of light at an intensity of 70 μmol m^-2^ s^-1^) in the growth chamber.

eGFP fluorescence in the inoculated cotyledons/hypocotyls and developing plantlets was observed every 3 weeks under the Leica MZ10F fluorescence stereomicroscope (Wetzlar, Germany). The frequency of cotyledons/hypocotyls-derived calli showing eGFP fluorescence was reported at 9 weeks after infection, as the [(number of eGFP fluorescent calli/total number of explants treated) × 100]. Transformation efficiency (TE) was calculated *in vitro* for each case study as the [(number of independent eGFP fluorescent shoots/total number of explants treated) × 100].

### 2.3 Genetic transformation efficiency of cotyledons and hypocotyls from flower-derived SEs in comparison with the meristematic bulk as starting explant

The genetic transformation experiment has been designed to evaluate the effect of the explant type on the regeneration of transgenic plants. With this aim, 100 MB slices of the table grape cultivar Thompson Seedless, obtained following the method described by Sabbadini et al., (2019b), together with 100 cotyledons and 100 hypocotyls of the same cultivar, have been inoculated with *A. tumefaciens strain* EHA105 expressing the *35S::eGFP::nptII* gene construct, following the agro-infection procedure described above. After co-culture all the explants were left to regenerate in the medium composed of MS basal salt and vitamins (Murashige and Skoog, 1962) including 30 g L^−1^ sucrose, 7 g L^−1^ plant agar, 13.2 µM BAP, 0.1 µM NAA, 146 µM kanamycin, 420 µM cefotaxime, and 475 µM carbenicillin.

eGFP fluorescence in the inoculated MBs, cotyledons, hypocotyls, and eventually in regenerating shoots was observed every 3 weeks under the stereomicroscope, as described above. The frequency of eGFP fluorescent calli and the transformation efficiency for each type of explant were reported and calculated as described above, at 9 weeks after infection.

### 2.4 Shoot elongation and plant acclimatization

Explants involved in both regeneration and transformation experiments were monitored for more than 9 weeks, transferring them to the same fresh culture medium every three weeks. At the third subculture, explants cultured on M1 were moved to elongation M1 supplemented with 1.1 μM BAP and 0.49 μM IBA as PGRs, while explants cultured on M2 were transferred to elongation M2 supplemented with 4.4 μM BAP. In the transformation trials, conducted following the same procedures, the culture media have been enriched with 146 µM kanamycin, 420 µM cefotaxime, and 475 µM carbenicillin.

Elongated shoots from the regeneration experiments, and elongated shoots showing stable eGFP green fluorescence from the transformation experiments, were isolated and transferred to PGR-free rooting medium consisting of half-strength Murashige and Skoog, 1962 salts and vitamins, supplemented with 15 g L^−1^ sucrose, 7 g L^−1^ plant agar including 146 µM kanamycin for the transformed plants. The acclimatization and the establishment of plantlets in the greenhouse were performed by transferring elongated and rooted *in vitro* plantlets into plastic propagation trays (60 holes) filled with commercial peat ensuring high humidity content, and then in pots of 14 centimetres in diameter containing commercial soil peat.

### 2.5 Molecular analysis of transgenic plants

#### 2.5.1 PCR analysis

The presence of the transgene in the selected regenerated lines was confirmed by PCR amplification of a portion of the 35S promoter present in the T-DNA sequence used in this study. DNA extraction from the putative transgenic lines was performed by using the Thermo Scientific Phire Plant Direct PCR Kit (Fisher Scientific), following the manufacturer’s instructions. Briefly, a 0.5 mm diameter leaf sample was collected by each plant line and placed into a tube containing 20 µL of the dilution buffer provided by the kit. After the processing of samples by grinding and vortexing, 1.25 µL were used as a template in a 50 µL PCR reaction. The PCR program consisted of an initial denaturation step at 98°C for 5 min, followed by 40 cycles of denaturation at 98°C for 5 s, annealing at 63.6°C for 5 s, and extension at 72°C for 20 s, and a final extension at 72°C for 1 min. PCR products were analysed by electrophoresis on 1% agarose gel stained with Invitrogen SYBR Safe (Fisher Scientific). The PCR products were visualized using a GelDoc EZ Imaging System (Bio-Rad). DNA amplicon for the analysis, spanning a 340 bp-long portion of the 35S, was obtained by PCR with the following forward (F) and reverse (R) primers: F 5′-CTTCGTCAACATGGTGGAGCACGACA-3′ and R 5′-TGGAGATATCACATCAATCCACTTG-3′. The positive control was represented by the DNA extracted from *Agrobacterium* plasmid containing the *35S*::*eGFP*::*nptII* transgenic cassette.

#### 2.5.2 Southern blot analysis of *in vivo* stabilized transgenic plants

The “Illustra DNA extraction kit PHYTOPURE” (GE Healthcare) was used to isolate genomic DNA starting from 1g of leaves by following the manufacturer’s instructions. Approximately ~ 15 µg of genomic DNA was digested with *KpnI* enzyme, separated on a 0.7% agarose gel at 4.5 V cm^-1^ and transferred on Hybond-N^+^ nylon membrane (GE Healthcare). The DNA probe (545 bp-long), corresponding to the *nptII* marker gene, was obtained by PCR with the following primers: F, 5’-CAGAGTCCCGCTCAGAAGAACTCGTCA-3’ and R, 5’-GGAAGGGACTGGCTGCTATTGGGCGAA-3’. The probe was labelled with [α-^32^P] dCTP using Random primer DNA labelling kit (Takara), and subsequently purified using Illustra ProbeQuant G-50 micro columns (GE Healthcare). The membrane was pre-hybridized for 1 h at 42°C in ULTRAhyb hybridization buffer (Ambion) containing 100 µg/mL of denatured UltraPure™ Herring Sperm DNA Solution (Thermo Scientific), then hybridized overnight at 42°C using of 10^6^ cpm mL^-1^ of labelled probe. Then, the filter was washed twice for 5 minutes in 2X SSC/0.1% SDS at 42°C and twice for 15 minutes in 0.1XSSC/0.1%SDS at 42°C and exposed to Carestream Kodak BioMax XAR films.

### 2.6 Statistical analysis

The regeneration and transformation experiments on cotyledons and hypocotyls were replicated three times. Data related to regeneration and transformation efficiencies were subjected to analysis of variance (ANOVA), with the type of explant, cultivar, and regeneration medium tested as sources of variation. The mean comparisons between the cultivar and regeneration medium were determined using the Student-Newman-Keuls t-test at p ≤0.05. All analyses were performed with the Statistica 7 software (Statsoft Tulsa, CA, USA). Data on the percentage of regeneration were transformed by the arcsine square root transformation, ARSIN (SQRT (X)) before analysis.

## 3 Results

### 3.1 *De novo* shoot organogenesis from cotyledons and hypocotyls of grapevine cultivar SEs

The new regeneration protocol from cotyledons and hypocotyls from the flower–derived SEs has been fine-tuned prior to performing genetic transformation experiments.

**Figure 2:**
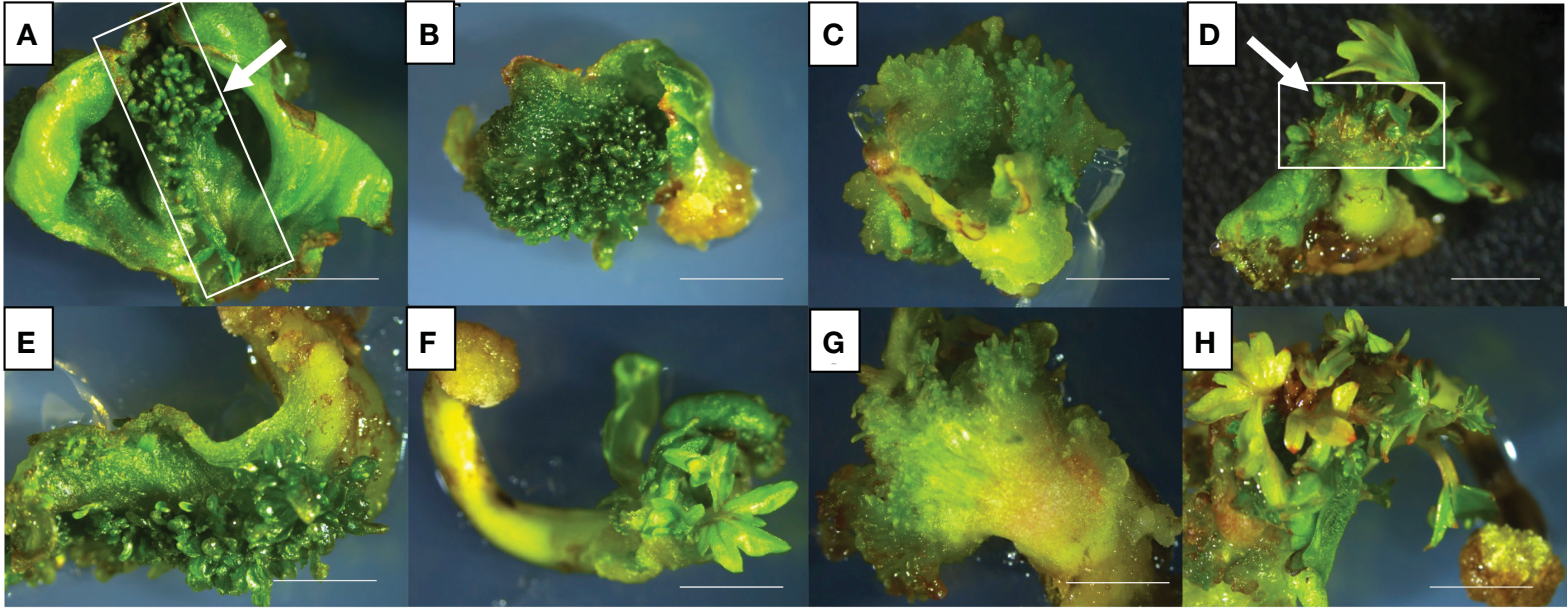
*De novo* adventitious shoot organogenesis from cotyledons **(A–D)** and hypocotyls **(E–H)** of Ancellotta **(A, E)**, Lambrusco Salamino **(B, F)**, and Thompson Seedless **(C, G)** after three weeks of culture. Shoot regeneration from cotyledons **(D)** and hypocotyls **(H)** of Lambrusco Salamino after 6 weeks on M1. The development and consequent proliferation of apical meristems, which prevailed over the adventitious regeneration of buds, were observed between the two cotyledonary leaves **(D)**. *De novo* shoot organogenesis on hypocotyls occurred at the distal end generally at the opposite side of the primary root **(H)** (*bars* = 5 mm).

In general, cotyledons of the three cultivars (Ancellotta, Lambrusco Salamino, and Thompson Seedless) showed a high regeneration efficiency when maintained for 3 weeks on MS culture media supplemented with both sets of hormones (BAP in combination with IBA, or BAP alone). On cotyledons, adventitious shoot regeneration occurred mainly on the adaxial surface along the central vein, while at a lesser extent in the region close to the area of conjunction with hypocotyl (**Figures 2A–D**). While hypocotyls induced adventitious shoot regeneration mainly from the distal end opposite to the radicle, especially at the wounded surface area of these explants (**Figures 2E–H**).

**Figure 3:**
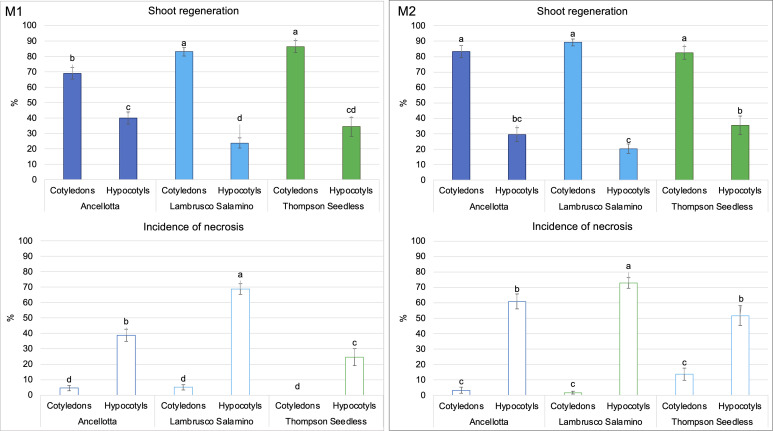
Shoot regeneration rate (%) and percentage of necrosis (%) of Ancellotta, Lambrusco Salamino, Thompson Seedless cotyledons, and hypocotyls cultured on regeneration M1 and M2, acquired after 9 weeks of culture. Means with different letters are significantly different according to the Student-Newman-Keuls (p ≤ 0.05) ± SE (n = 50). Error bars represent the standard errors of three replications.

The apical meristems of SEs, located between cotyledons, also underwent cell proliferation, leading to the development of well-formed auxiliary buds, which were not always clearly distinguishable from those developed adventitiously directly from the cotyledonary leaves (**Figure 2D**). *De novo* shoot organogenesis frequently occurred from cotyledons nearby the midvein (**Figure 2A**), and from hypocotyls in the area close to their cut distal ends (**Figure 2H**). The regeneration efficiency, in terms of explants able to produce at least one adventitious shoot, and the incidence of necrosis, expressed as the number of necrotic explants on the total explants cultured, were recorded at the end of the third subculture, after 9 weeks of culture (**Figure 3**). Between the two types of organs from the flower-derived SEs, cotyledons were the most regenerative starting explants, reaching a value higher than 70% for each genotype cultured on both culture media (**Figure 3**). This regeneration capacity varied depending on the cultivar. By using M1 (supplemented with 4.4 μM BAP and 0.49 μM IBA), significant statistical differences among the different cultivars were detected. In particular, Ancellotta was the less responsive (69%) compared to the other two cultivars, which reached 83% (Lambrusco Salamino) and 86% (Thompson Seedless) of regeneration efficiency. Cotyledons of the three cultivars grown on M2 (supplemented with 13.2 μM BAP) showed a similar high regeneration efficiency, around 80-90%. Hypocotyls, on the other hand, resulted in a reduced regeneration efficiency, less than 40% in all the cultivars and on both media tested. Indeed, many hypocotyls failed to initiate shoot regeneration, necrotizing during the 9 weeks of culture, especially for Lambrusco Salamino explants, 70% of which necrotized. The percentage of necrotized hypocotyls was lower when grown on M1, especially for Ancellotta and Thompson Seedless cultivars, similar to the incidence of necrotization observed when using cotyledons.

Each type of explant (cotyledons and hypocotyls) was able to regenerate shoots in each of the two culture media, but with different percentages of regenerating explants and explant regeneration capacity (number of regenerated adventitious shoots). In fact, the highest average number of shoots per explant was recorded when both flower-derived SEs’ explants of Thompson Seedless were cultured on M2 (more than five shoots/explant from cotyledons and four shoots/explant from hypocotyls). Ancellotta and Lambrusco Salamino hypocotyls regenerated a higher number of shoots per explant compared to cotyledons, in both culture media, while PGRs type and concentration used did not significantly affect shoot production in these two cultivars (**Table 1**).

**Table 1:** Effect of culture medium on the average number of shoots regenerated from cotyledons and hypocotyls of Ancellotta, Lambrusco Salamino, and Thompson Seedless.

Genotype	Type of explant	M1^*^	M2^*^
Ancellotta	Cotyledons	1.80 ± 0.15^cd^	1.40 ± 0.26^cd^
	Hypocotyls	2.75 ± 0.34^c^	1.77 ± 0.21^cd^
Lambrusco Salamino	Cotyledons	0.73 ± 0.08^cd^	0.61 ± 0.08^d^
	Hypocotyls	1.55 ± 0.07^cd^	1.28 ± 0.06^cd^
Thompson Seedless	Cotyledons	1.37 ± 0.12^cd^	5.71 ± 0.72^a^
	Hypocotyls	1.38 ± 0.17^cd^	4.23 ± 0.58^b^

Data were recorded 9 weeks after culture initiation on media 1 (M1) and 2 (M2).

^*^The values represent the means (± SE) of three independent experiments. Mean values within the column followed by the same letter are not significantly different by Student Newman-Keuls post hoc test (p ≤ 0.05).

**Figure 4:**
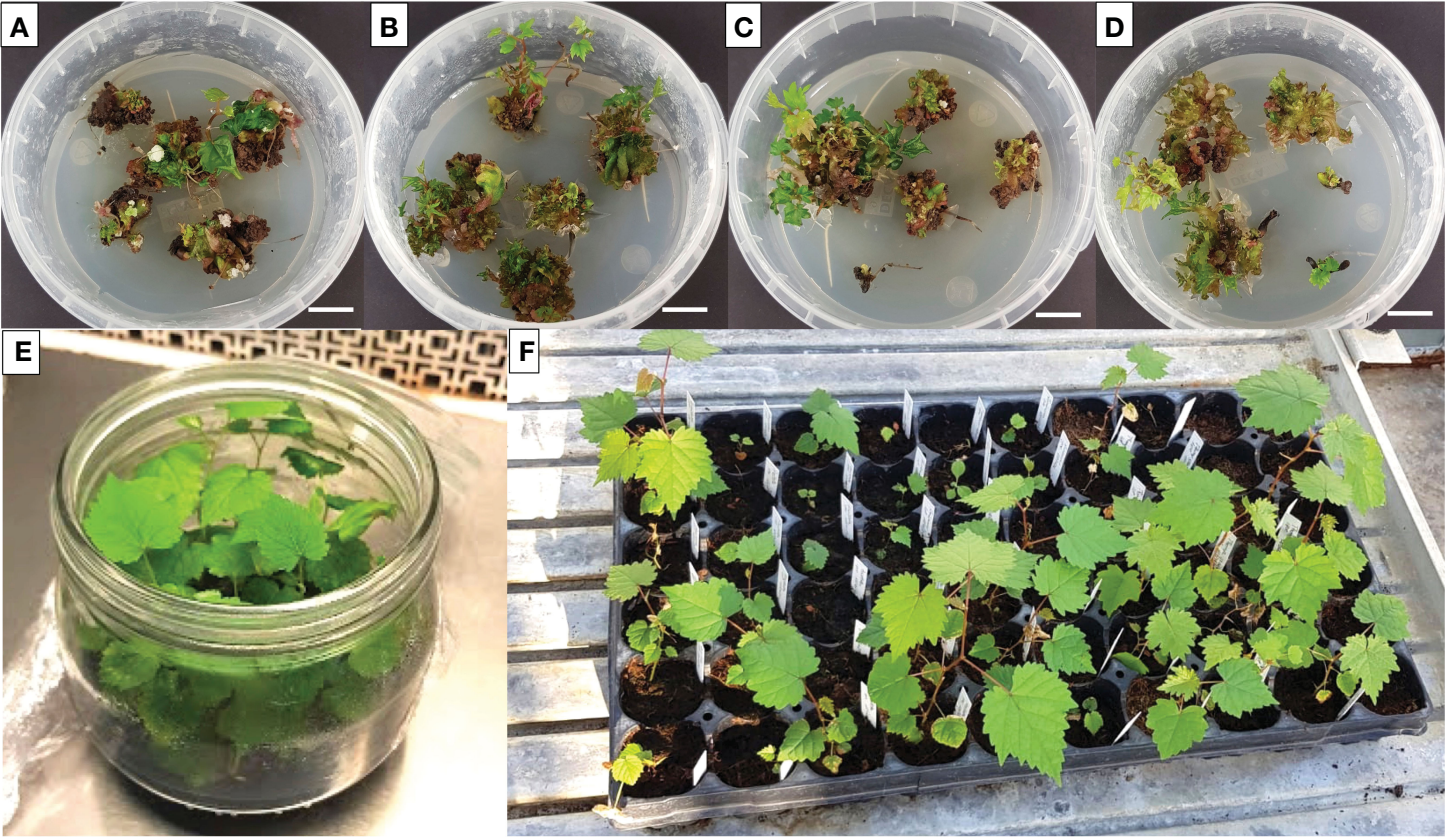
Representative image of Lambrusco Salamino shoot proliferating structures derived from the culture of cotyledons **(A, B)**, and hypocotyls **(C, D)**, on M1 **(A, C)**, and M2 **(B, D)**, each one consisting of halved cytokinin concentration, after twelve weeks of culture (*bar* = 1 cm). Isolated shoots were left to grow in a PGR-free MS medium for root initiation and shoot elongation **(E)** followed by acclimatization in the greenhouse **(F)**.

After nine weeks of culture, only the regenerating explants were transferred to a fresh regeneration medium containing a halved concentration of cytokinins compared to the initial substrate composition. At 12 weeks, the initial explants (cotyledons, and hypocotyls) were no longer distinguishable, as they developed callus-like structures characterized by continuous shoot regeneration (**Figures 4A–D**). Elongated shoots from these new structured explants were isolated and grown on MS medium without PGRs to stimulate root induction (**Figure 4E**), which occurred after four weeks. Subsequently, the *in vitro* shoots that developed roots were successfully acclimatized (around 85%, data not shown), giving rise to plants with the typical appearance and conformation of the reference cultivar (**Figure 4F**).

### 3.2 Genetic transformation efficiency of cotyledons and hypocotyls of the three grapevine cultivars cultured on the two regeneration/selection media

**Figure 5:**
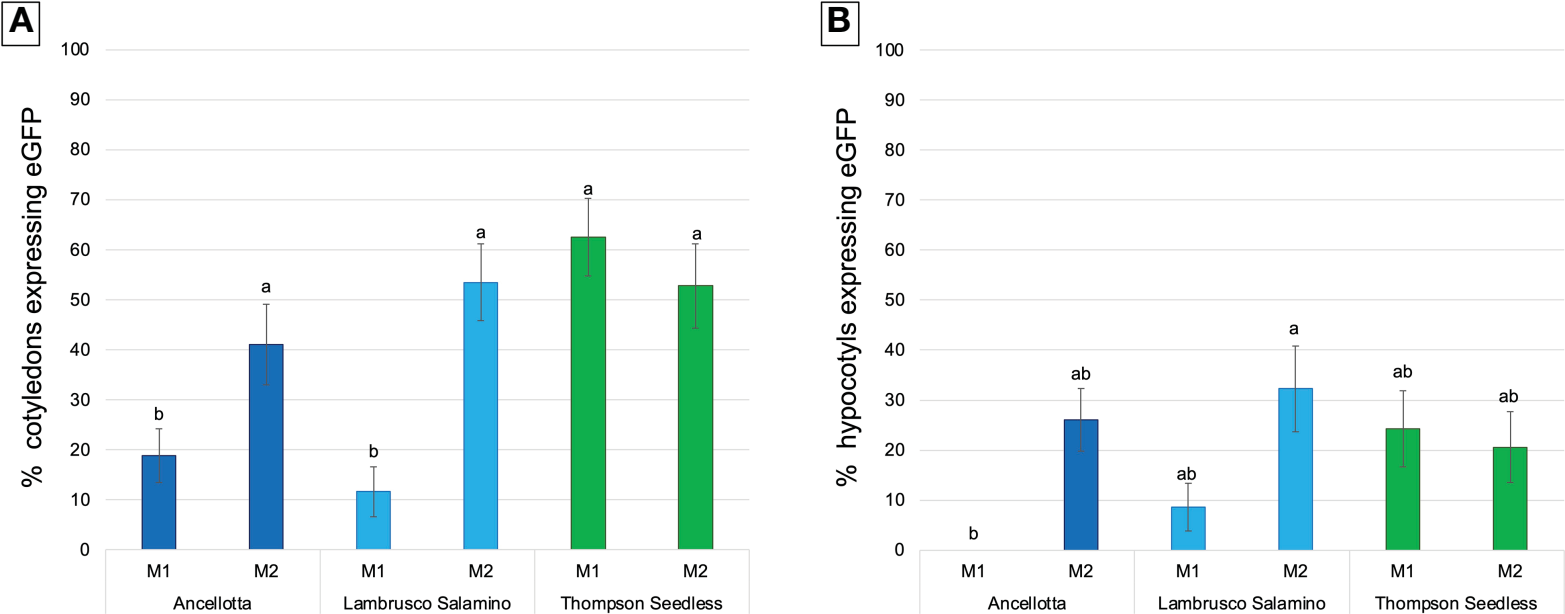
Frequency (%) of calli expressing eGFP fluorescence obtained from cotyledons **(A)** and hypocotyls **(B)** of Ancellotta, Lambrusco Salamino, Thompson Seedless under UV light on the total number of treated explants, at 9 weeks after infection. These explants were cultured on regeneration/selection media M1 and M2 supplemented with 146 µM kanamycin, 420 µM cefotaxime, and 475 µM carbenicillin. Means with different letters are significantly different according to the Student-Newman-Keuls (p ≤ 0.05) ± SE (n=50).

For transformation studies, the regeneration protocols described above for cotyledons and hypocotyls of Ancellotta, Lambrusco Salamino, and Thompson Seedless were tested to verify their efficiency in generating new transformed plants after *Agrobacterium-*mediated transformation experiments. The response to genetic transformation was first evaluated after 9 weeks of tissues cultivation on the two selection media, each supplemented with 146 µM kanamycin, 420 µM cefotaxime, and 475 µM carbenicillin, by the identification of the number of cotyledons and hypocotyls that developed green fluorescent calli lines and expressed as the percentage of the total explants treated (**Figure 5**).

Cotyledons of the three cultivars exerted higher regeneration competence rather than hypocotyls in both media tested, as observed in the previous regeneration experiments.

**Figure 6:**
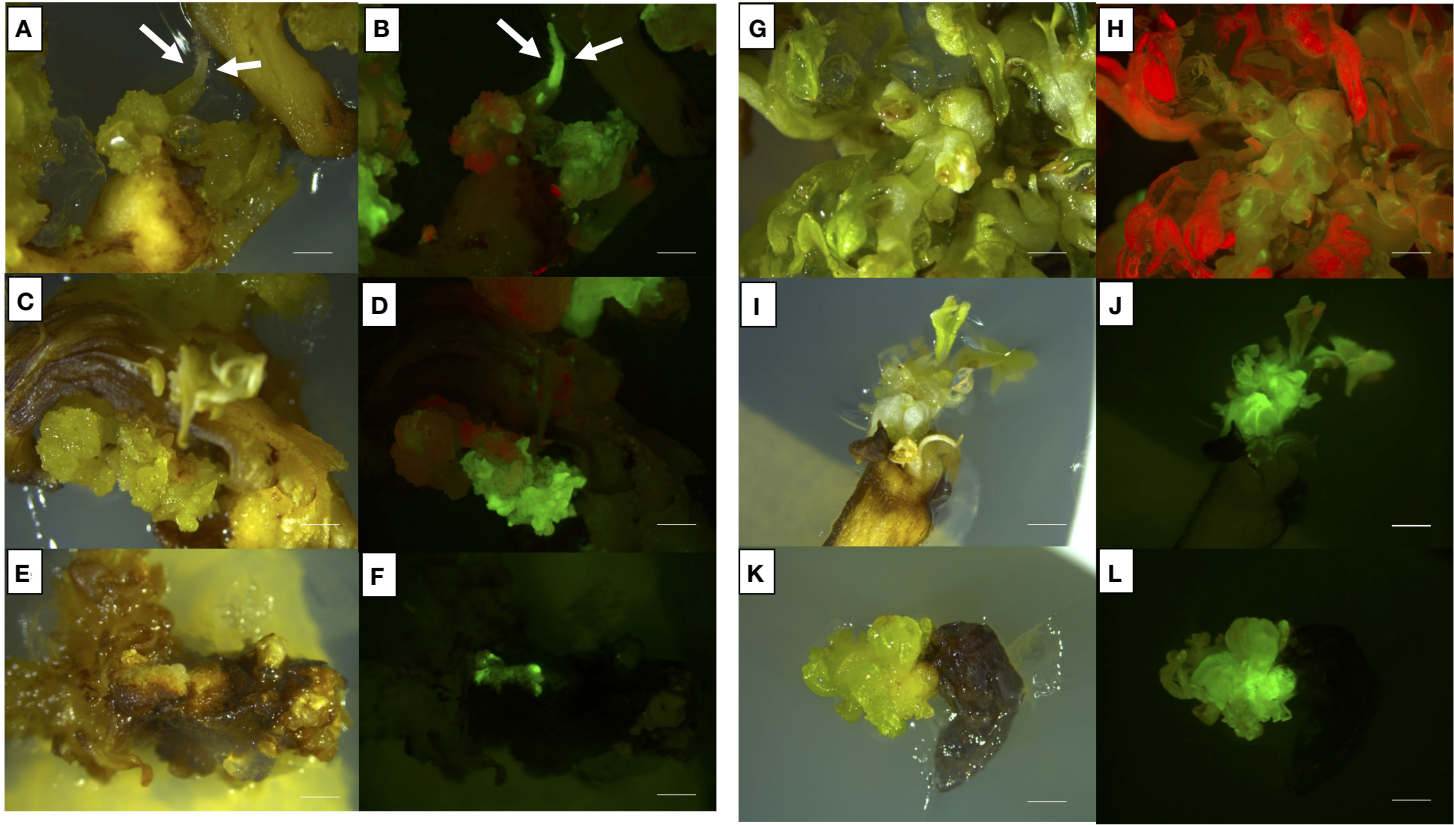
Representative images of shoots or shoots-derived structures developed after 9 weeks from agro-infection on Thompson Seedless **(A–D)**, Lambrusco Salamino **(E, F)**, and Ancellotta **(G, H)** cotyledons, and Thompson Seedless hypocotyls **(I–L)**, illuminated by white light **(A, C, E, G, I–K)**, and UV light **(B, D, F, H, J–L)**. **(A, B)** refer to Thompson Seedless cotyledons cultured on M1; **(C, D)** refer to Thompson Seedless cotyledons cultured on M2; **(E, F)** refer to Lambrusco Salamino non-organogenic calli regenerated on M2 from cotyledons; **(G, H)** refer to Ancellotta cotyledons cultured on M2; **(I–J)** refer to Thompson Seedless hypocotyls cultured on M1; **(K–L)** refer to Thompson Seedless hypocotyls cultured on M2 (*bar* = 2 mm).

During the 9 weeks of selection, only some callus areas maintained a high regenerative capacity, while the other parts became progressively necrotic, probably for the action of the selective agent, thus they were progressively discarded. Cotyledons of Thompson Seedless produced the highest percentage of eGFP fluorescent calli when cultured on M1 (62.5%), comparable with the data obtained from the culture of cotyledons in M2 (52.8%) (**Figure 5A**). For hypocotyls, M1 was also slightly more efficient than M2, although there was not statistical difference in terms of eGFP fluorescent calli, settlings on values between 20% and 24% (**Figure 5B**). When Thompson Seedless was cultured on M1 (4.4 μM BAP and 0.49 μM IBA), 6 (12% TE) and 3 (6% TE) independent eGFP fluorescent lines regenerated from cotyledons (**Figures 6A, B**) and hypocotyls (**Figures 6I, J**), respectively. While 7 (14% TE) and 6 (12% TE) fluorescent Thompson Seedless independent lines regenerated from cotyledons (**Figures 6C–F**) and hypocotyls (**Figures 6K, L**), respectively, when grown on M2 (13.2 µM BAP). Roots emitting fluorescence, directly regenerated from the explants, were also observed in Thompson Seedless cotyledons cultured on M1 (highlighted with arrows in **Figures 6A, B**). Ancellotta cotyledons and hypocotyls showed variable genetic transformation responses depending on the choice of selection/regeneration medium. For both explants, M1 had a lower efficiency in generating eGFP fluorescent calli after *A. tumefaciens*-mediated transformation experiment compared to the efficiency displayed in M2 (**Figures 5A, B**). Although cotyledons and hypocotyls in M2 reached a level of frequency of eGFP fluorescent calli at 9 weeks comparable to that observed for Thompson Seedless (41% and 26% in cotyledons and hypocotyls, respectively), most of these fluorescent calli failed to regenerate adventitious shoots.

From this set of experiments, it was possible to isolate a single eGFP fluorescent adventitious shoot derived from Ancellotta cotyledons cultured on M2. However, this selected adventitious shoot did not have a uniform distribution of fluorescence, resulting in a chimeric form (**Figures 6G, H**). With the aim to generate a new non-chimeric transgenic line of Ancellotta, this chimeric line was transferred to fresh M2 with a higher concentration of the selective agent (208.6 µM kanamycin), to increase selective pressure and promote cell proliferation of transformed cells only. After four months of culture, new Ancellotta shoots fully fluorescent under UV light were isolated and grown to give rise to new uniform fluorescent adventitious shoots. Jointly with what was observed in Ancellotta, also in Lambrusco Salamino the culture medium containing the highest cytokinin concentration (M2) allowed to significantly increase the number of calli showing green fluorescence, both on cotyledons and hypocotyls, compared to the same explants placed in M1 (**Figure 5**). On M2, among the 53.5% of cotyledons and 32.3% of hypocotyls which showed eGFP fluorescence, only two Lambrusco Salamino green-fluorescent calli developed and proliferated in the selective media, which failed to regenerate eGFP fluorescent shoots (**Figures 6E, F**). These calli appeared white, friable, and characterized by generally disorganized growth, and their continued subculture in the same fresh media, or on media with higher auxin content (having only 2.5 µM IBA) did not promote any shoot development. After the third sub-culture, when new shoots of Ancellotta and Thompson Seedless were visible from the green-fluorescent calli (**Figures 6B, D, F, H, J, L**), they were selected and transferred to two different selection media enriched with the same auxin concentration, but with a reduced cytokinin content (1.1 µM BAP together with 0.49 µM IBA for explants cultured on M1, and 4.4 µM BAP for explants cultured on M2), maintaining 146 µM kanamycin concentration for the whole process. Finally, only elongated shoots expressing eGFP were cultured on a kanamycin-enriched PGR-free MS medium to stimulate *in vitro* root emergence *in vitro*.

In total, nine putative transgenic Thompson Seedless lines and one Ancellotta line, derived from cotyledons and hypocotyls, were successfully acclimatized in plastic pots, and grown in a dedicated greenhouse.

### 3.3 Meristematic bulk slices, cotyledons, and hypocotyls responses after genetic transformation in the model cultivar Thompson Seedless

Thompson Seedless cultivar is known for the high transformation efficiency obtainable by using sliced portions of MBs as described by Mezzetti et al. (2002) and Sabbadini et al. (2019b). With the aim to identify the most efficient genetic transformation protocol, the MB protocol was compared with the new protocol based on the use of cotyledons and hypocotyls obtained from flower–derived SEs. All the explants (MBs, cotyledons, and hypocotyls) were inoculated with the same gene construct used in the previous transformation experiment (*35S*::*eGFP*::*nptII*). The selection of putatively transformed events occurred in the same regeneration/selection medium optimized for MBs in a previous study (Sabbadini et al., 2019b), which has a composition very similar to M2 with the addition of 0.01 μM 1-Naphthaleneacetic acid (NAA).

**Figure 7:**
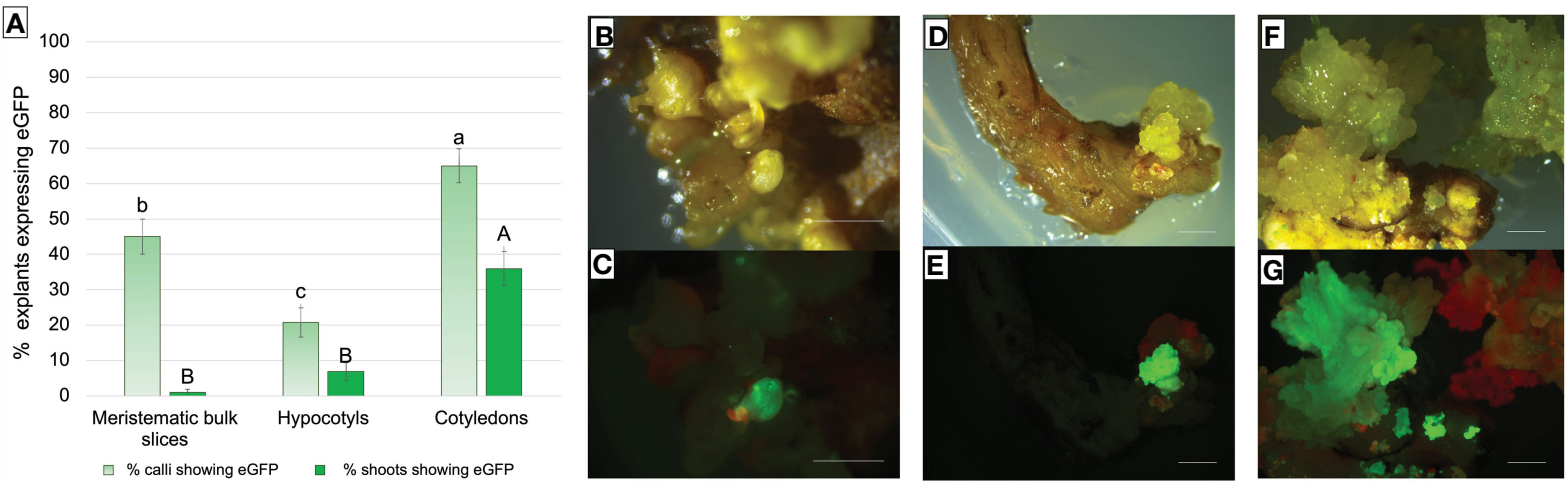
**(A)** Frequency (%) of Thompson Seedless explants-derived calli and shoots expressing eGFP fluorescence under UV light on the total number of treated explants. eGFP fluorescence was recorded after 9 weeks of selection on MB slices, hypocotyls, and cotyledons of the Thompson Seedless cultivar transformed with *35S*::*eGFP*::*nptII* gene construct. Small letters represent differences in eGFP fluorescence among the different explants in terms of calli that showed at least one fluorescent dot on their surface; capital letters represent differences in eGFP fluorescence between the various explants in terms of shoots showing eGFP. Means with different letters are significantly different according to the Student-Newman-Keuls (p ≤ 0.05) ± SE (n=100). **(B–G)** Representative images of shoots or shoots-derived structures developed after 9 weeks of culture on regeneration/selection medium supplemented with 146 µM kanamycin, 420 µM cefotaxime, and 475 µM carbenicillin using Thompson Seedless MB slices **(B, C)**, hypocotyls **(D, E)**, and cotyledons **(F, G)**, illuminated by white light **(B, D, F)**, and UV light **(C, E, G)** (*bar* = 2 mm).

About 20% of hypocotyls-, 40% of MBs-, and 60% of cotyledons-derived calli showed eGFP fluorescence at 9 weeks after infection (**Figure 7A**).

Differences were found in the efficiency of regeneration of fluorescent shoots and their development. Cotyledons showed the highest significant transformation efficiency in terms of explants displaying eGFP fluorescent shoots, compared to MB slices and hypocotyls explants (**Figure 7A**). Although MB slices had on average a significantly higher number of calli showing eGFP fluorescence than hypocotyls, the number of regenerated shoots showing eGFP fluorescence was higher in hypocotyls. A total of 1, 7, and 26 regenerating fluorescent shoots were obtained from MBs (**Figures 7B, C**), hypocotyls (**Figures 7D, E**), and cotyledons (**Figures 7F, G**), respectively. In addition, contrary to what was observed on MB slices, cotyledons and hypocotyls were able to regenerate more than one putative transgenic shoot from the same explant (data not shown).

### 3.4 Molecular confirmation of transgene integration

**Figure 8:**
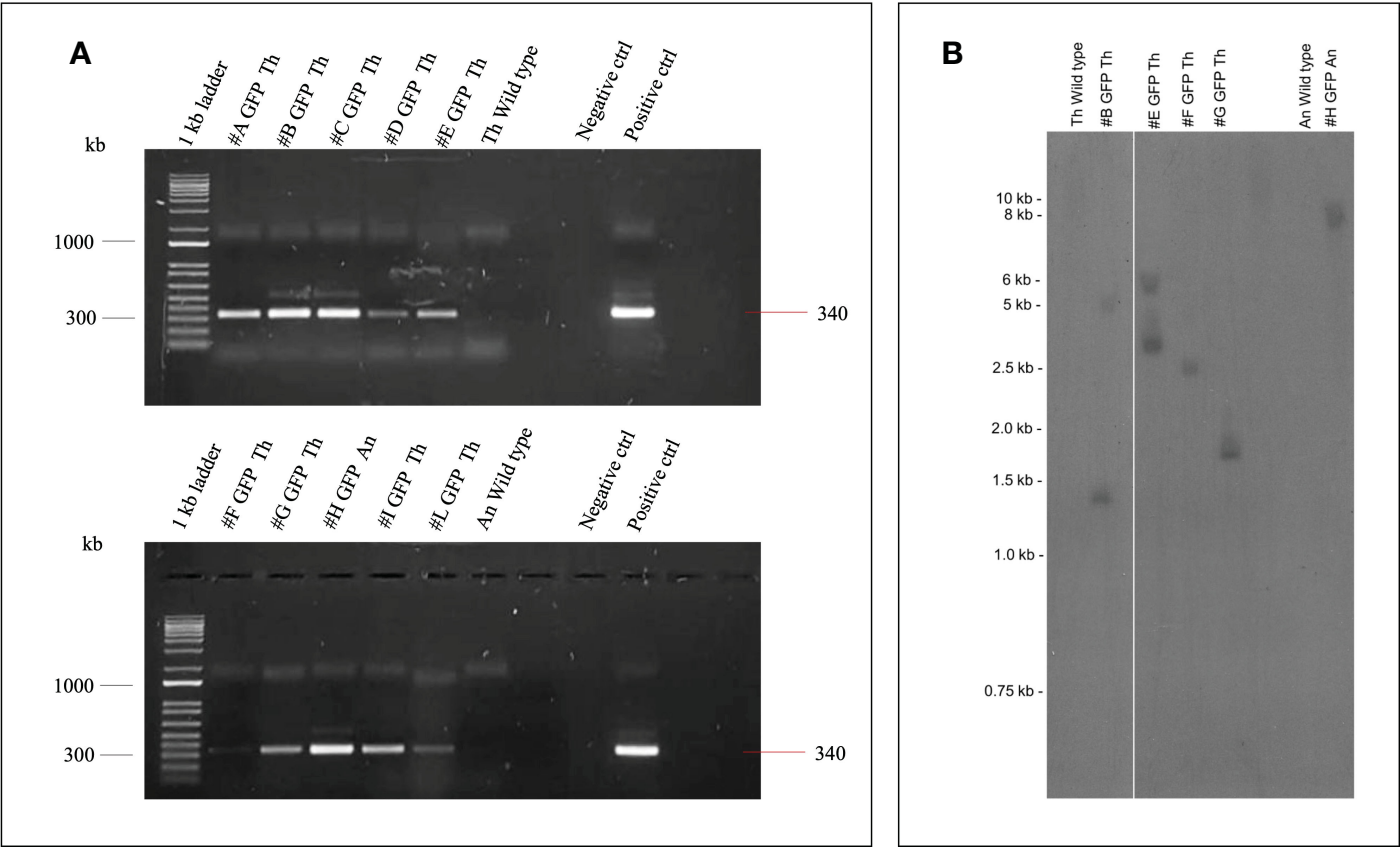
**(A)** Amplification of 35S promoter (340 bp) gene fragment from genomic DNA of nine transgenic lines (#A - #L) of Thompson Seedless (Th), one line (#H) of Ancellotta (An) obtained after *A*. *tumefaciens* infection, plus the wild-type controls. Positive control corresponds to *pK7WG2*-*35S-eGFP-nptII* PCR product. The lanes labelled “Negative control” show the PCR result using deionized sterile water. 1Kb ladder (Invitrogen™ by Thermo Fischer scientific). **(B)** Southern blot analysis of *nptII* copy number in grapevine transgenic plants. Genomic DNA from Thompson Seedless and Ancellotta wild-type and transgenic lines was digested with *KpnI*, which cuts T-DNA at a single position.

PCR analysis was performed on the first nine regenerated Thompson Seedless and one Ancellotta independent lines selected for eGFP to confirm their transgenic state. In all the ten transgenic lines analysed, amplification of the expected 340 bp DNA amplicon, corresponding to a fragment of the 35S promoter, was confirmed (**Figure 8A**).

The transgenic state of a subset of randomly chosen transformed Thompson Seedless lines (#B, #E, #F, and #G) and of the Ancellotta line (#H) was checked by Southern blot analysis using *nptII* as probe (**Figure 8B**). The analysis confirmed that the Thompson Seedless transgenic lines originated from independent transformation events.

## 4 Discussion

SEs are the final results of a specific developmental pathway that can be asexually induced *in vitro*, with a morphology practically identical to that of zygotic embryos, and with the agronomical advantage of being obtained from somatic tissues (Faure et al., 1996; Correia et al., 2016). Recent and past research works concerning the stable genetic transformation of grapevine cultivars and rootstocks, aiming at transferring selectable marker and reporter genes, were primarily focused on the somatic embryogenesis regeneration system, mainly using pro-embryogenic masses (Dhekney et al., 2008), embryogenic calli (Iocco et al., 2001; Torregrosa et al., 2002; López-Pérez et al., 2008; Nakajima et al., 2020), embryogenic cell suspension cultures (Wang et al., 2005), and SEs (Li et al., 2001; Li et al., 2006; Li et al., 2008; Dhekney et al., 2009; Dhekney et al., 2016) as starting explants. In fact, embryonic tissues are characterized by a great regeneration competence, which can be exploited to maintain morphogenic potential for several years (Martinelli et al., 2001a). In woody fruit plants such as apple and pear, sections of cotyledons and/or hypocotyls derived from zygotic embryos, were used for adventitious shoot induction (Korban and Skirvin, 1985; Browning et al., 1987). However, similar experiments on other woody plants are scarce, probably due to the high level of heterozygosity and the need to cultivate clones identical to those typical of the selected crop variety (Capriotti et al., 2020). For this reason, SEs, obtained following the newly established protocols on Ancellotta, Lambrusco Salamino, and Thompson Seedless grapevine cultivars (Capriotti et al., 2022), were sectioned by separating cotyledons from hypocotyls, and used as starting explants in this study to optimize regeneration and *Agrobacterium*-mediated transformation protocols *via* organogenesis. Despite the regeneration from cotyledons and hypocotyls of mature SEs having been attempted in previous studies for other grapevine genotypes (Vilaplana and Mullins, 1989; Martinelli et al., 2001b), this is the first report in which both type of explants were efficiently employed to induce high regeneration. Prior to *Agrobacterium*-mediated transformation of Ancellotta, Lambrusco Salamino, and Thompson Seedless, the regeneration from cotyledons and hypocotyls was fine-tuned in two MS-based culture media, characterized by different auxin and cytokinin ratios. We found that the highest regeneration efficiencies (from 69% up to 89%) were obtained when cotyledons were used as starting explants irrespective of the culture medium, albeit good regeneration rates (from 20.3% up to 40.1%) were also obtained for hypocotyls from all the three genotypes. Our study determined that both cotyledons and hypocotyls were proper starting tissues for stimulating the production of adventitious shoots, in contrast with the findings by Vilaplana and Mullins (1989), who did not observe shoot regeneration from cotyledons when cultured on NN-based media with the same type and concentrations of PGRs. In similar previous research, 18% of Chardonnay cotyledons formed shoots, and less than 50% of Thompson Seedless, Grenache, and Gloryvine hypocotyls or whole SEs regenerated adventitious shoots (Vilaplana and Mullins, 1989; Martinelli et al., 2001b). Therefore, in this work we innovatively reported cotyledon and hypocotyl regeneration in Ancellotta and Lambrusco Salamino, and the significant improvement of Thompson Seedless regeneration potential. Although similar regeneration responses were observed between the two types of media, M2 characterized by a higher concentration of BAP, had a significant effect on the number of shoots produced only in Thompson Seedless. Again, as in studies conducted on regeneration from leaves and nodal segments of woody plant species, we emphasized the role of BAP as the most efficient cytokinin to induce direct shoot regeneration (Khan et al., 2015; Kumsa and Feyissa, 2019; Ricci et al., 2020). As reported by Vilaplana and Mullins (1989), we observed the continuous induction of adventitious shoots from the explants, which appeared during the first stage of development as round green buds producing the first plantlets after only 40 days from culture initiation (**Figures 2D, G**).

Embryo dormancy and low embryo-plantlet conversion rate are known to be major bottlenecks to the *in vitro* culture of SEs. These major drawbacks could be bypassed by the application of this innovative regeneration strategy *via* organogenesis, avoiding plant losses, and increasing the regeneration potential in all the three grapevine cultivars, which showed comparable high competence to regenerate adventitious shoots.

The regeneration protocols fine-tuned in this study were then applied to obtain transgenic grapevine plants *via Agrobacterium*-mediated transformation. The TE was calculated by recording eGFP fluorescence in both explants-derived calli and shoots. The cotyledons and hypocotyls resulted valid in terms of regeneration of green fluorescent calli. However, M2, having a higher concentration of cytokinin, was more effective in inducing the highest percentage of fluorescent calli derived from cotyledons and hypocotyls of Ancellotta and Lambrusco Salamino.

In accordance with Sabbadini et al., (2019b), regardless of the regeneration potential, genotype has a great influence in determining the transformation competence. Indeed, in this study, when cotyledons or hypocotyls of the Ancellotta cultivar were subjected to agro-infection with the *35S-eGFP-nptII* gene construct, eGFP fluorescence was detected in about 40% for the first, and 25% for the second type of explant, respectively, but the regeneration of only one transformed shoot was obtained from these explants. To our knowledge, this is the first transformation result on Ancellotta, obtained from an adventitious shoot with a chimeric expression of eGFP fluorescence. This confirms that chimerism remains a major problem in establishing genetic transformation protocols for different grapevine cultivars (Franks et al., 2002; Dalla Costa et al., 2019). However, in this work we demonstrate that the regeneration of a new stable transgenic line is possible from a chimeric explant through the continuous culture in media having having high concentration of cytokinin and increased concentration of the selective agent.

For Lambrusco Salamino, eGFP fluorescent calli were observed, but they turned necrotic and did not induce the regeneration of any transformed shoot. These results suggest that the protocol still needs proper modifications, in terms of medium composition, and selection procedure, in order to optimize these latter steps for this cultivar as well. The potency of this regenerative method was evident in the Thompson Seedless model genotype, allowing the obtainment of nine transgenic lines selected from M1, and 13 from M2. Moreover, the Southern blot analysis suggests that the proposed transformation method gives rise to a low number of T-DNA insertions, as observed in previous Thompson Seedless lines obtained from MB slices (Mezzetti et al., 2002; Sabbadini et al., 2019b).

Although many scientific works have amply demonstrated the potential of somatic embryogenesis as regeneration tool to achieve high genetic transformation efficiencies, the use of explants such as pro-embryogenic masses and embryogenic calli requires their continuous production through a new induction of somatic embryogenesis or secondary embryogenesis, thus following generally lengthy procedures. In addition, these small explants easily undergo necrosis and it is generally necessary to adapt the transformation protocol to the genotype used (Torregrosa et al., 2002; López-Pérez et al., 2008).

However, also SEs following the somatic embryogenesis system are not always the most performant acceptor explants, as reported by Dai and co-authors, who were not able to efficiently transform embryogenic calli and somatic embryos since they were subjected to necrosis, obtaining successful results only using pro-embryogenic masses of Chardonnay (Dai et al., 2015).

**Figure 9:**
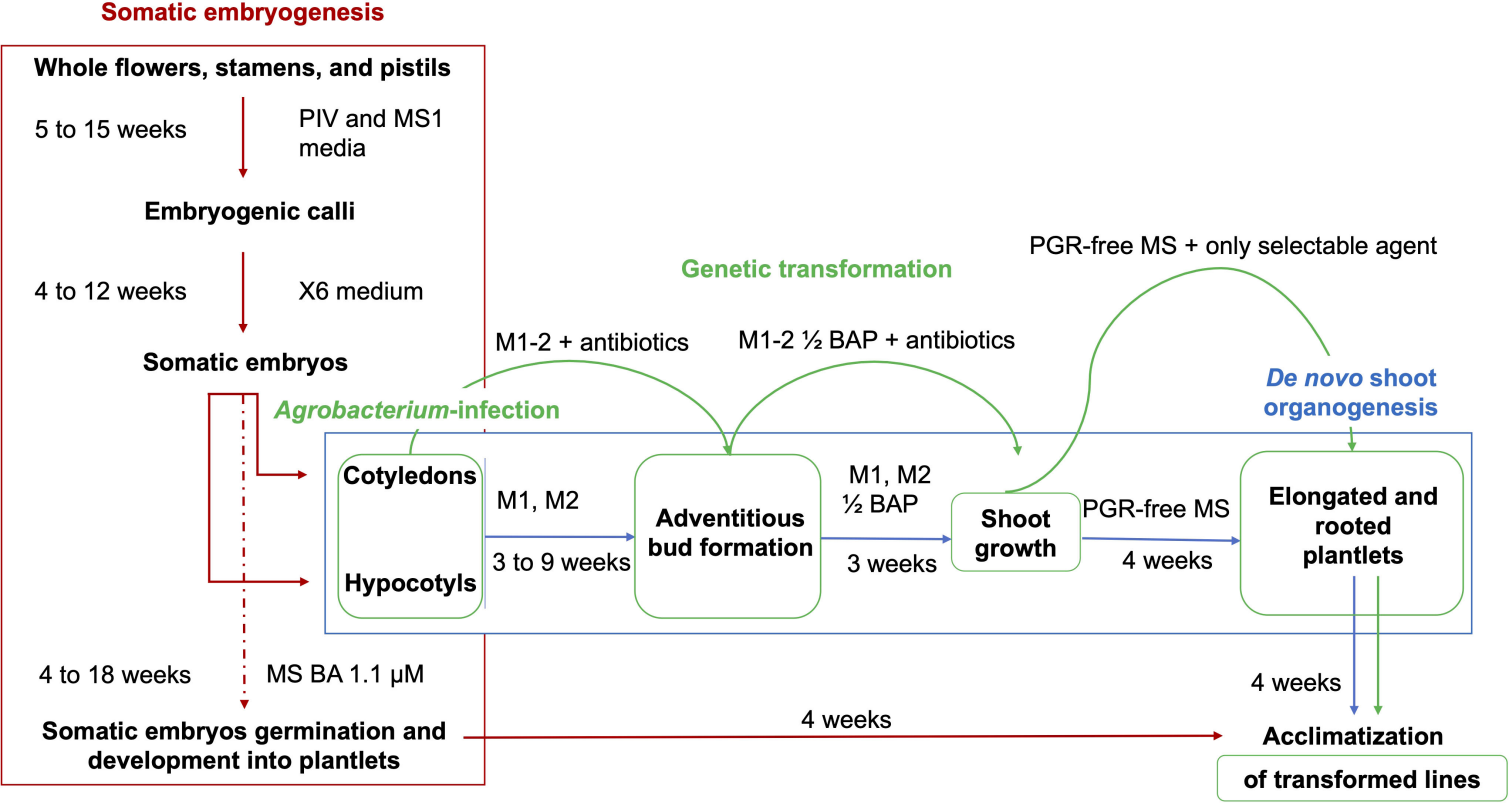
Schematic representation of the regeneration methods and *Agrobacterium*-mediated transformation protocols from cotyledons and hypocotyls obtained from flower-derived somatic embryos of grapevine cultivars. Embryogenic calli are obtained from the culture of whole flowers, stamens, and pistils on PIV and MS1 media. These calli are continuously proliferated on X6 medium, with the production of somatic embryos at the cotyledonary stage. The somatic embryogenesis regeneration pathway is interrupted through the isolation of cotyledons and hypocotyls from the somatic embryos, which are separated and induced to form adventitious shoots on appropriate regeneration media (M1 and M2). Shoot development occurs in media with halved BAP concentrations, while the elongation and the emergence of roots are stimulated from the shoots in a PGR-free MS medium, which is followed by the acclimatization in the greenhouse. Agro-infected cotyledons and hypocotyls chase the same steps for the stimulation of *de novo* shoot formation, in media supplemented with 420 µM cefotaxime, 475 µM carbenicillin, and 146 µM kanamycin.

The novel regeneration strategy described in this study (summarized in **Figure 9**) represents a valid alternative in grapevine genetic transformation scenario, that can also be extended and adapted to several recently studied genotypes of *Vitis* species, for which the direct transformation of SEs could still remain ineffective (Carra et al., 2016; Forleo et al., 2021; Capriotti et al., 2022).

Regarding Thompson Seedless, it has been demonstrated that MBs have a quite high efficiency in producing new transgenic plants (Mezzetti et al., 2002; Sabbadini et al., 2019b), however, the *in vitro* regeneration and transformation protocols based on cotyledons and hypocotyls allowed the enhancement of the number of fluorescent shoots produced in this model cultivar. The same protocol also contributed to obtain a new transgenic line of the recalcitrant cultivar Ancelotta, therefore, it can be considered of interest to be applied for other recalcitrant grapevine genotypes.

## 5 Conclusions

Several factors are involved in defining the competence to regenerate new organs, and among them, the type of starting explant, genotype, type of culture medium, PGRs, and their concentrations seem to be crucial in ensuring success both in the regeneration and subsequent proliferation of plant material, and in genetic engineering approaches. Through this study, somatic embryogenesis was used as a regenerative technique that allowed the production of a very high number of somatic explants in the form of cotyledons and hypocotyls. Such explants, having regained juvenile characteristics, retain a high propensity for regeneration as well as good transformation efficiency. Specifically, cotyledons were the most responsive somatic embryo-derived explant in the two substrates tested, and the increase in cytokinin content in the medium had a positive effect on the number of regenerated shoots only in the Thompson Seedless cultivar, while, during the nine weeks following the genetic transformation, the presence of 13.2 μM BAP in the selective medium, increased the number of cotyledons and hypocotyls that showed eGFP fluorescence on explants derived from Ancellotta and Lambrusco Salamino. Furthermore, the genetic transformation of three different types of explants such as MB slices, cotyledons, and hypocotyls derived from SEs, demonstrated that the cotyledons are the most efficient starting tissue for the cultivar Thompson Seedless, and the only type of explant capable of leading to the regeneration of a genetically transformed shoot in the Ancellotta cultivar. These novel *in vitro* regeneration and genetic transformation protocols on fruit plants such as grapevine, in its wine grape and table grape varieties, are as essential as ever for the application of new and emerging modern biotechnologies aimed at promoting tolerance to pathogens and pests, increasing resilience to abiotic stresses, and improving agronomic traits such as productivity, fruit quality, and seedlessness (Sabbadini et al., 2021; Nerva et al., 2023).

## Data availability statement

The original contributions presented in the study are included in the article/supplementary material. Further inquiries can be directed to the corresponding author.

## Author contributions

LC, SS, and BMe conceived and designed the study. LC made the experiments, performed the statistical analysis, and prepared the original draft of the manuscript. SS, AR, and IP contributed to the regeneration and transformation experiments and plant acclimatization. BMo and TP carried out and interpreted the molecular analyses, wrote specific parts of the article. All authors contributed to the article and approved the submitted version.
